# The Efficacy and Safety of High Dose (10 mg) of Desloratadine (Dazit® 10) in the Treatment of Chronic Spontaneous Urticaria in India: A Phase III, Multicentric, Open-Label, Single-Arm Study

**DOI:** 10.7759/cureus.53125

**Published:** 2024-01-28

**Authors:** Saurabh Kapadia, Siddabathuni Nageswaramma, Keyur Shah, Ajit Singh, Satyaprakash C Mahajan, Ajay Deshpande, Sayantani Chakraborty, Bikash R Kar, Pinjala Padmaja, Subhash C Bharija, Maulik Doshi, Pravin Ghadge, Mukesh Gabhane, Shruti Dharmadhikari, Amey Mane, Suyog Mehta

**Affiliations:** 1 Department of Dermatology, Kanoria Hospital & Research Centre, Gandhinagar, IND; 2 Department of Dermatology, Guntur Government General Hospital, Guntur, IND; 3 Department of Dermatology, Medilink Hospital & Research Center, Ahmedabad, IND; 4 Department of Allergy and Pulmonary Medicine, Sawai Man Singh (SMS) Hospital, Jaipur, IND; 5 Department of Dermatology, Supe Heart and Diabetes Hospital and Research Centre, Nashik, IND; 6 Department of Dermatology, Oyster & Pearl Hospital, Pune, IND; 7 Department of Dermatology, R. G. Kar Medical College and Hospital, Kolkata, IND; 8 Department of Dermatology, Institute of Medical Sciences (IMS) and SUM Hospital, Bhubaneswar, IND; 9 Department of Dermatology, Osmania General Hospital, Hyderabad, IND; 10 Department of Dermatology, Sir Ganga Ram Hospital, Delhi, IND; 11 Department of India Clinical Research, Sun Pharma Laboratories Limited, Mumbai, IND; 12 Department of Medical Affairs and Clinical Research, Sun Pharma Laboratories Limited, Mumbai, IND

**Keywords:** up-dosing, quality of life (qol), dosing, urticaria activity score, oral antihistamine, chronic spontaneous urticaria (csu), uas7, desloratadine, antihistamine, csu

## Abstract

Background: Chronic spontaneous urticaria (CSU) is a debilitating affliction that affects diverse quality of life (QoL) parameters such as sleep, self-esteem, and daily activities. Second-generation antihistamines, such as desloratadine, are more effective and safer in managing CSU. Desloratadine is a nonsedating, potent, and highly selective H1 receptor antagonist. At its daily dose of 5 mg, almost half of CSU patients do not show symptomatic improvement. European Academy of Allergy and Clinical Immunology (EAACI)/Global Allergy and Asthma European Network (GA2LEN)/European Dermatology Forum (EDF) (EuroGuiDerm)/Asia Pacific Association of Allergy, Asthma and Clinical Immunology (APAAACI) guidelines recommend increasing the dosage to up to four times in such nonresponsive patients. However, there is insufficient clinical evidence in Indian settings.

Method: We evaluated the efficacy and safety of 10 mg desloratadine (OD) in 256 nonresponsive patients with moderate to severe CSU. The primary outcome was the change in Urticaria Activity Score (UAS7) from baseline to four weeks. Additionally, change in Chronic Urticaria Quality of Life (CU-Q2oL) scores during the course of treatment was also evaluated.

Result: The mean UAS7 scores showed a significant reduction from 31.9 ± 4.8 at baseline to 18.2 ± 8.1 at the end of the study (p < 0.0001). The use of a higher dose of desloratadine also decreased the CU-Q2oL scores significantly from 59.8 ± 14.7 at baseline to 35.4 ± 10 at four weeks (p < 0.0001). The incidence of adverse events (AEs) possibly linked to the drug was low (1.6%), and no serious adverse events were reported.

Conclusion: Results indicated improvements in the disease severity as well as its positive impact on participants’ QoL. This study confirms the efficacy and safety of daily use of a twofold dose of desloratadine in nonresponsive moderate to severe CSU patients.

## Introduction

Chronic urticaria is a dermatological condition characterized by the presence of recurrent and pruritic wheals (raised, red skin blotches), angioedema (swelling in deeper skin layers), or both for six weeks or longer. If there is no obvious causal trigger, it is called chronic spontaneous urticaria (CSU), previously known as chronic idiopathic urticaria [1,2]. The global point prevalence of CSU is 0.5%-1% of the general population, with the disease duration ranging from one to five years, but it is likely to last for decades in severe cases [3,4]. Due to a lack of large studies, the prevalence of CSU in the Indian context is unavailable [3]. However, the point prevalence of chronic urticaria in Asia is reported to be 1.4%, which is much higher than that in Europe (0.5%) and North America (0.1%) [5]. Recently published data suggest that it is a common disease with increasing prevalence, which is as common in children as it is in adults, with some studies reporting an even higher prevalence in children [4,5]. It is now well established that it affects females twice as it affects males [6]. CSU is debilitating and distressing primarily due to excessive itch, pain, and the unappealing appearance of lesions. This affects various health-related quality of life (QoL) parameters such as sleep, mental status, self-esteem, social interactions, and even daily leisure activities [7]. The extent of impairment is similar to that seen in patients with chronic cardiac disease, with reports suggesting stress, emotional disturbance, and other psychiatric issues to be very common in CSU patients [8,9].

The skin lesions in CSU are a result of skin mast cell degranulation, which causes a release of histamine, proteases, various cytokines, and prostaglandin-like mediators. These products lead to vasodilation, increased vascular permeability, and sensory nerve stimulation causing swelling, redness, and itch in the affected area [10]. Since histamine is the key player in CSU pathogenesis, antihistamines have long been used as a treatment option. Second-generation antihistamines have shown to be more efficient and safer than older drugs and are now the first line of treatment for patients with CSU [1,11].

Desloratadine, one of the important second-generation drugs and the active component of the widely used drug loratadine, is a selective, potent, and nonsedating H1 receptor antagonist [12]. It additionally inhibits the release of other inflammatory products secreted by basophils, eosinophils, and mast cells through mechanisms that are independent of H1 receptor antagonism [13]. Due to these properties, desloratadine at its conventional dose of 5 mg is reported to alleviate the objective as well as subjective measures of CSU and has been rated high by patients and healthcare providers in various clinical trials [12-14]. Despite treatment with effective H1 antihistamines, only <50% of CSU patients have improvement in their symptoms with the standard dose [6]. The European Academy of Allergy and Clinical Immunology (EAACI)/Global Allergy and Asthma European Network (GA2LEN)/European Dermatology Forum (EDF) (EuroGuiDerm)/Asia Pacific Association of Allergy, Asthma and Clinical Immunology (APAAACI) guidelines strongly recommend increasing the dosage of antihistamine up to fourfold in patients not responding to the conventional dosage [1]. A similar recommendation has been made in the recent Skin Allergy Research Society guidelines [3]. Despite expert recommendations, there is insufficient clinical evidence available for up-dosing [15-18].

As there was a lack of data regarding up-dosing of desloratadine in Indian settings, we performed a phase III study to evaluate the efficacy and safety of desloratadine 10 mg once a day (OD) in patients with moderate to severe CSU who did not respond to the conventional 5 mg OD dose.

## Materials and methods

Study design

**Figure 1:**
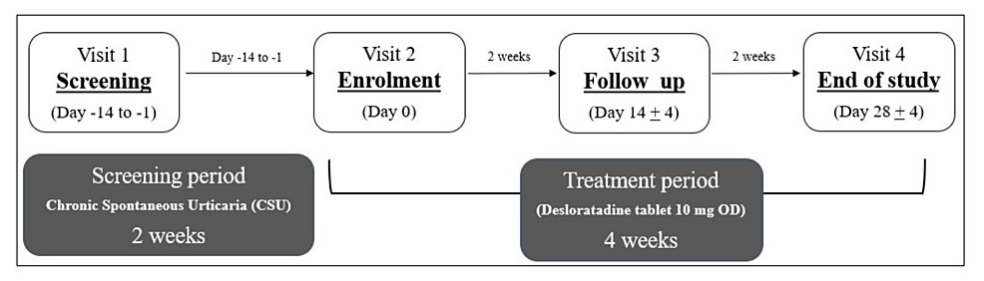
Study schematics of the phase III, single-arm, non-randomized, open-label trial of daily dose of 10 mg desloratadine in chronic spontaneous urticaria patients CSU: chronic spontaneous urticaria

This was a phase III, single-arm, non-randomized, open-label study done across 10 sites throughout India (protocol number: ICR/16/004, CTRI/2017/10/010146). All patients gave written informed consent before screening for eligibility. The study period was six weeks, which included two weeks of screening and four weeks of treatment as depicted in Figure 1. Treatment with 10 mg desloratadine began on the day of enrolment. The study had four scheduled visits, during which a questionnaire related to quality of life (QoL) was administered, participants’ well-being and drug compliance were monitored, and the patient-reported disease record diary was assessed for completion. On the first and last visits, blood and urine samples were also collected for routine laboratory investigations.

Study population (inclusion/exclusion criteria)

Eligible participants were adults (male and female) aged between 18 and 65 years with a history of CSU since at least six weeks prior to screening, an active CSU since at least three weeks prior to screening with wheals present for >3 days/week, and nonresponders to 5 mg desloratadine (taken daily since at least two weeks before screening). Participants were enrolled if they had moderate to severe CSU at the time of screening as well as three days before enrolment. Moderate CSU was defined as the presence of 20-50 wheals/24 hours (wheal and pruritus score = 2), and severe CSU was defined as the presence of >50 wheals/24 hours or large confluent areas of wheals (wheal and pruritus score = 3). Participants were excluded if they had acute or inducible urticaria, uncontrolled asthma, symptomatic seasonal/perennial rhinitis, allergies that are manifested as rash, or any immunological condition other than CSU or were hospitalized due to worsening of CSU three months prior to screening. They were also excluded if they had other clinically significant conditions that made them unfit to participate, were involved in any other clinical trial 60 days before screening, or were using any other antihistamine (except desloratadine 5 mg once daily) within two weeks prior to their screening. Pregnant and lactating women were also excluded from the study.

The study was approved by the institutional/independent ethics committee of all participating sites (approval numbers: 3477/MC/EC/2017, Arc/55/5596, D4R/IMS-SH/S0A|170068, ЕС/11/17/1288, ECR/467/Inst/AP/2013/RR-16) and was conducted in accordance with the Declaration of Helsinki (2013) and Good Clinical Practice (GCP) guidelines.

Treatment design

This was a single-arm, open-label study, and thus, no blinding/unblinding procedure was followed. Desloratadine (10 mg) (Dazit® 10; manufactured by Sun Pharma) tablets were provided by Sun Pharma Laboratories Limited. It was taken by the participants once daily, with or without food. Subjects who consumed at least 80% of the assigned investigational product for the specified duration (each visit) and completed the evaluation within the designated visit with no major protocol violations were considered treatment compliant.

Measurement of outcomes

The primary outcome of the study was changes in the weekly Urticaria Activity Score (UAS7) from baseline to the end of the study (four weeks). The secondary outcomes included changes in UAS7 from baseline to two weeks, changes in Chronic Urticaria Quality of Life (CU-Q2oL) scores from baseline to two and four weeks, and safety assessments.

Efficacy Assessments

Evaluation of efficacy was done by recording UAS7. UAS7 is a patient-reported score that includes wheal and pruritus scores, ranging from 0 to 3 (none to intense). This score, summed over a week, gives the weekly score values (0 to 42), with higher scores representing severe symptoms. UAS7 score-based health states were defined as follows: urticaria-free = 0, well-controlled urticaria = 1-6, mild urticaria = 7-15, moderate urticaria = 16-27, and severe urticaria = 28-42 [19]. Participants were provided with a diary to record their responses every day.

Another tool that was used to evaluate efficacy was the CU-Q2oL score. This is a standard questionnaire covering various aspects of the participants’ lives, such as sleep, behavior, appearance, and activities. The effect of CU on each selected item is scored on a four-point scale (1 = no effect at all, 5 = very much). This scale reflects the quality of life of CU patients, where a decreased score reflects improvement in the QoL of CU patients [20]. This was administered during each participant visit.

Safety Assessments

Safety assessments included monitoring and reporting of all adverse events (AEs) and severe adverse events (SAEs), their severity, their onset, and their relation with the administered study drug. Other than these, physical health and vital sign evaluation (body temperature, pulse rate, sitting blood pressure, respiratory rate, etc.) were done at each visit. On the first and last visits, additional hematological tests (complete blood count, and renal and liver function tests), 12-lead electrocardiogram, and routine urine analysis were also done for all participants.

Statistical analyses

Assessment of primary efficacy outcome was performed on the intention-to-treat (ITT) population, which included all the enrolled participants. Other populations on which analyses were performed included the following: safety population, which included all enrolled participants who received at least one dose of study medication; per protocol (PP) population, which included participants who completed the study according to the protocol without major deviations; and modified intent-to-treat (mITT) population, which included all enrolled participants who met all the inclusion/exclusion criteria, were administered at least one dose of assigned product, and returned for at least one post-baseline evaluation visit.

Descriptive variables were presented as mean and standard deviation (SD) or as median, range, and 95% confidence interval. Categorical variables were summarized as count and percentage. To test for changes in the primary and secondary efficacy outcomes, a paired t-test was used at a two-sided 5% level of significance using the Statistical Analysis System (SAS) version 9.1.3 or higher (SAS Institute Inc., Cary, NC). Missing data were handled using the last observation carried forward method.

## Results

Participant disposition and demographic characteristics

A sample size of 191 participants was calculated to detect a difference in means of 1.00 at 90% power, assuming that the common standard deviation is 3.00 using a two-group t-test with a 0.05 two-sided significance level. Accounting for an attrition rate of 15%, the required sample size was calculated to be 225.

**Figure 2:**
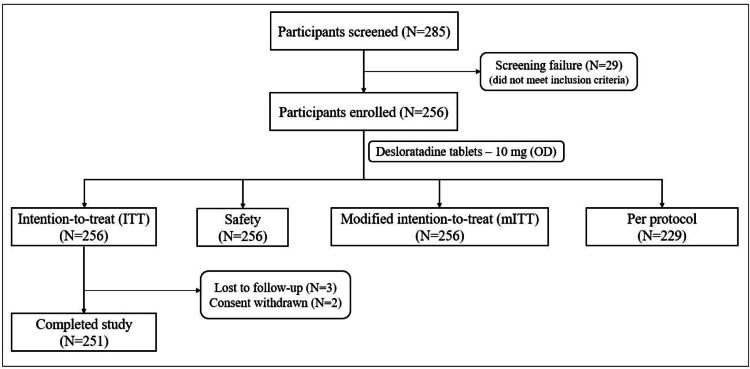
Participant disposition (CONSORT) CONSORT: Consolidated Standards of Reporting Trials, ITT: intention-to-treat, mITT: modified intent-to-treat

Between December 4, 2017, and September 7, 2018, 256 participants were enrolled in the study across 10 sites and were considered as ITT, safety, and mITT populations. The per protocol category had 229 participants, as around 17% of the participants had protocol deviations. A protocol deviation was defined as nonadherence to protocol-specific study procedures or schedules that did not involve inclusion/exclusion criteria, primary objective variable criteria, and/or GCP guidelines. Deviations were minor and did not impact the study. After four weeks of drug intervention, 251 (98%) participants completed the study. Of the five (2%) participants who did not complete the study, three were lost to follow-up and two withdrew their consent (Figure 2). The baseline characteristics of the study population are presented in Table 1.

**Table 1:** Demographic characteristics at baseline ITT: intention-to-treat, UAS7: weekly Urticaria Activity Score, CU-Q2oL: Chronic Urticaria Quality of Life, SD: standard deviation, Min: minimum value, Max: maximum value, BMI: body mass index, CSU: chronic spontaneous urticaria

Characteristics (ITT population)	Statistics (N = 256)		
	Number (percent)	Median (min, max)	Mean + SD
Gender			
Female	134 (52.3%)		
Male	122 (47.7%)		
Age (years)		33 (18, 64)	35.3 + 11.1
Weight (kg)		60 (35.3, 85)	60.8 + 8.3
Height (cm)		158 (138, 188)	158.9 + 8.3
BMI (kg/m^2^)		24.2 (15.8, 40.2)	24.2 + 3.5
Severity of CSU based on UAS7			
Moderate	31 (12.1%)		
Severe	225 (87.9%)		
Number of wheals (per 24 hours)		40 (15,70)	42.4 + 11.4
Frequency of wheals (days/week)		5 (3, 7)	5.2 + 1.2
Non-satisfaction with 5 mg desloratadine			
Yes	256 (100%)		
No	0		
UAS7		31 (20.5, 42)	31.9 + 4.8
CU-Q2oL score		58.5 (27, 109)	59.8 +14.7

Gender distribution was almost equal in the study, with 52.3% of the participants being females. The mean age of the enrolled participants was 35.3 + 11.1 years. Based on UAS7 scores, a majority of the enrolled participants had severe CSU (N = 225; 87.9%), and the rest had a moderate form of CSU (N = 31; 12.1%). The mean number of wheals recorded in 24 hours was 42.4, and their mean frequency was around five days per week. No participant was satisfied with his/her current treatment regimen of 5 mg desloratadine that he/she took at least two weeks prior to enrolment. Their UAS7 scores ranged between 20.5 and 42, with a mean value of 31.9, which indicated severe urticaria. The mean CU-Q2oL score was 59.8, indicating a high degree of quality of life impairment.

By the end of the study, 251/256 (98%) participants showed compliance of at least 80% to their assigned drug and were considered as study drug compliant.

Efficacy evaluation

Primary Efficacy Evaluation

The mean UAS7 values after four weeks of treatment with 10 mg desloratadine showed a 42.3% reduction from its baseline values (18.2 versus 31.9; p < 0.0001 from baseline) (Table 2), and this reduction was statistically as well as clinically significant. It indicated an overall drop in the extent of severity of CSU from severe to moderate category.

**Table 2:** Change in UAS7 from baseline to four weeks after treatment (intention-to-treat population) Change from baseline = post-treatment values - baseline values, p-value: Wilcoxon signed-rank test UAS7: weekly Urticaria Activity Score, SD: standard deviation, min: minimum value, max: maximum value, NA: not applicable

Statistics	UAS7 visit 2 (baseline)	UAS7 visit 4 (four weeks)	Change in UAS7 from baseline
Number	256	251	251
Mean + SD	31.9 + 4.8	18.2 + 8.1	-13.8 + 9
Median (min, max)	31 (20.5, 42)	19 (0, 42)	-12.5 (- 41, 4.5)
% change in mean	NA	NA	-42.3%
p-value	NA	NA	<0.0001

Secondary Efficacy Evaluation

This objective included the evaluation of change in UAS7 from baseline to first two weeks of treatment and change in the CU-Q2oL from baseline to two and four weeks of treatment with 10 mg desloratadine.

UAS7 values showed a significant reduction of 20% after just two weeks of treatment with 10 mg desloratadine. The mean weekly scores decreased from 31.9 at baseline to 25.5 after two weeks, thus decreasing the population severity of CSU to moderate (Table 3).

**Table 3:** Change in UAS7 from baseline to two weeks after treatment (intention-to-treat population) Change from baseline = post-treatment values - baseline values, p-value: Wilcoxon signed-rank test UAS7: weekly Urticaria Activity Score, SD: standard deviation, min: minimum value, max: maximum value, NA: not applicable

Statistics	UAS7 visit 2 (baseline)	UAS7 visit 3 (two weeks)	Change in UAS7 from baseline
Number	256	255	255
Mean + SD	31.9 + 4.8	25.5 + 7.2	-6.4 + 6.4
Median (min, max)	31 (20.5, 42)	28 (0, 42)	-5 (-31, 8)
% change in mean	NA	NA	-20%
p-value	NA	NA	<0.0001

**Figure 3:**
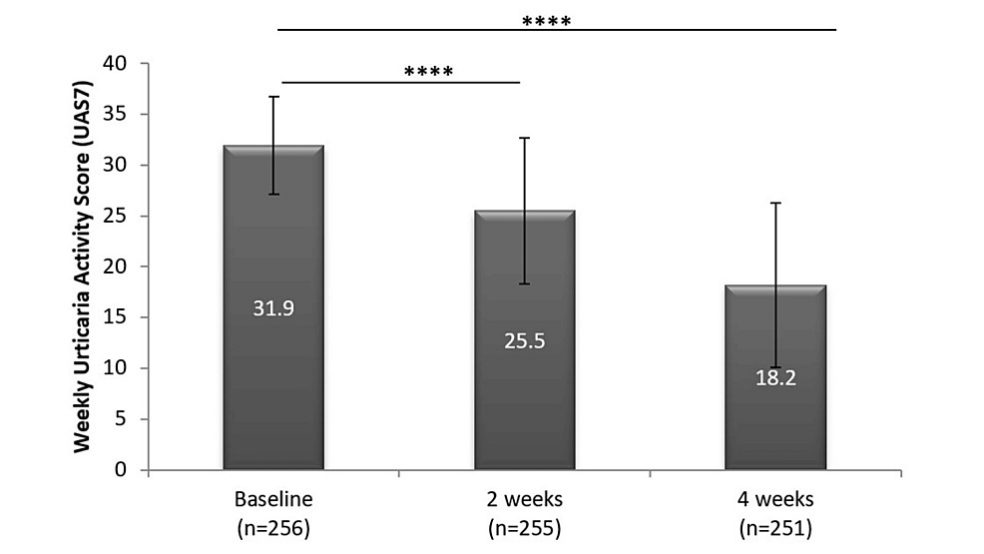
Significant reduction in UAS7 score following daily treatment with 10 mg desloratadine (mean ± SD plotted for each time point) ****p < 0.0001 UAS7: weekly Urticaria Activity Score, SD: standard deviation

As shown in Figure 3, UAS7 shows a significant reduction from baseline to subsequent weeks.

The proportion of patients with severe CSU, as estimated by the UAS7 scoring categories, also reduced with four weeks of 10 mg desloratadine treatment. At baseline, 225 participants had severe CSU, and by the end of the study, more than half of them (59.1%) had moved to the moderate category. Similarly, for the 31 participants who had moderate CSU at baseline, almost half of them (51.1%) showed improvement in disease symptoms and moved to the mild CSU category. Table 4 shows the proportion of participants in each category two and four weeks after treatment with 10 mg desloratadine.

**Table 4:** Severity of CSU in participants by UAS7 at day 14 and day 28 after treatment UAS7 score classification: severe urticaria = 28-42, moderate urticaria = 16-27, mild urticaria = 7-15, well-controlled urticaria = 1-6, urticaria-free = 0 CSU: chronic spontaneous urticaria, UAS7: weekly Urticaria Activity Score

Day 0 (number (%))	Day 14 (number (%))					Day 28 (number (%))				
	Severe	Moderate	Mild	Well-control	Urticaria-free	Severe	Moderate	Mild	Well-control	Urticaria-free
Severe (225 (100%))	133 (59.1%)	74 (32.9%)	15 (6.7%)	1 (0.4%)	1 (0.4%)	29 (12.9%)	133 (59.1%)	38 (16.9%)	12 (5.3%)	10 (4.4%)
Moderate (31 (100%))	0 (0%)	22 (71%)	9 (29%)	0 (0%)	0 (0%)	0 (0%)	9 (29.3%)	16 (51.6%)	1 (3.2%)	3 (9.7%)

**Figure 4:**
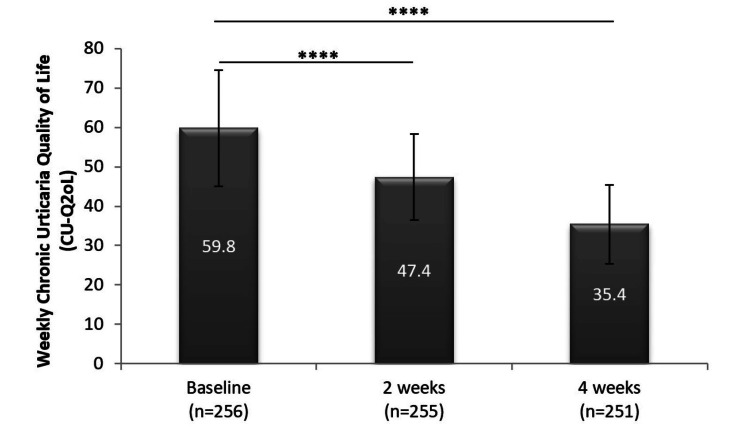
Significant reduction in Cu-Q2oL score following daily treatment with 10 mg desloratadine (mean ± SD plotted for each time point) ****p < 0.0001 CU-Q2oL: Chronic Urticaria Quality of Life, SD: standard deviation

The CU-Q2oL showed a significant 19.2% drop from the baseline scores after just two weeks of treatment (59.8 versus 47.4; p < 0.0001) with 10 mg desloratadine, and after another two weeks of treatment, this reduction doubled to 38.5% (59.8 versus 35.4; p < 0.0001) (Table 5, Figure 4).

**Table 5:** Change in weekly CU-Q2oL from baseline to two and four weeks after treatment (intention-to-treat population) Change from baseline = post-treatment values - baseline values, p-value: Wilcoxon signed-rank test CU-Q2oL: Chronic Urticaria Quality of Life, SD: standard deviation, min: minimum value, max: maximum value, NA: not applicable

Statistics	Visit 2 (baseline)	Visit 3 (two weeks)	Visit 4 (four weeks)
CU-Q2oL			
Number	256	255	251
Mean + SD	59.8 + 14.7	47.4 + 10.9	35.4 + 10
Median (min, max)	58.5 (27, 109)	46 (24, 83)	33 (23, 83)
Change in CU-Q2oL from baseline			
Number	NA	255	251
Mean + SD		-12.4 + 10.6	-24.6 + 16
% change in mean		-19.2%	-38.5%
Median (min, max)		-11 (-52, 40)	-23 (-77, 34)
p-value		<0.0001	<0.0001

Safety evaluation

During the study period, a total of 15 AEs were reported, which affected 5.9% of the total participants. The majority of these (6 (2.3%)) were unlikely related to the study drug, five (2%) events were possibly related, and four (1.6%) events were unrelated. Of the total AEs, 12 were reported to be mild in nature, and three were reported to be moderate. A majority of the adverse events were mild gastrointestinal disorders such as nausea, vomiting, and dry mouth in five (2%) participants. The details of other AEs are presented in Table 6.

**Table 6:** Severity of adverse events in the participants *One patient had two different events. AE: adverse event

System organ class	Total number of events	Number (%) of participants (mild)	Number (%) of participants (moderate)
Number of subjects with at least one AE	15	11* (4.3%)	3 (1.2%)
Gastrointestinal disorders	5	5 (2 %)	0 (0%)
Dry mouth		2 (0.8%)	0 (0%)
Nausea		1 (0.4%)	0 (0%)
Vomiting		2 (0.8%)	0 (0%)
General disorders and administration site conditions	5	3 (1.2%)	2 (0.8%)
Asthenia		2 (0.8%)	0 (0%)
Pyrexia		1 (0.4%)	2 (0.8%)
Infections and infestations	1	0 (0%)	1 (0.4%)
Upper respiratory tract infection		0 (0%)	1 (0.4%)
Nervous system disorders	1	1 (0.4%)	0 (0%)
Headache		1 (0.4%)	0 (0%)
Reproductive system and breast disorders	2	2 (0.8%)	0 (0%)
Premenstrual pain		2 (0.8%)	0 (0%)
Respiratory, thoracic, and mediastinal disorders	1	1 (0.4%)	0 (0%)
Oropharyngeal pain		1 (0.4%)	0 (0%)

All reported AEs resolved during the study course.

No clinically significant changes were noted during the vital sign examination and physical examination of the participants. Clinical laboratory data, including hematology and biochemistry parameters, as well as the electrocardiogram (ECG) of the participants showed no clinically important changes. Isolated abnormal values with clinical significance were reported as adverse events. There were no serious adverse events during the course of the study.

## Discussion

The use of second-generation antihistamines is the gold standard for the treatment of CSU due to their higher efficacy and safety. Cetirizine, levocetirizine, fexofenadine, loratadine, bilastine, desloratadine, and rupatadine are few of the examples of routinely used drugs for this purpose [1]. Desloratadine has been added to this list for about two decades and is now being considered a first-line therapy for patients with chronic urticaria. A high avidity and specificity toward the H1 receptor, linear pharmacokinetics, and lack of side effects such as sedation and cognitive impairment all render desloratadine a preferred treatment option in the management of chronic urticaria [13,21,22]. It has been used across several clinical trials in CSU patients at its conventional dose of 5 mg/day and has been extensively described to be effective as well as safe [12-14,23,24]. However, more than half of the patients have been reported to not respond to the daily standard dose of second-generation antihistamines [6]. A study of 390 patients with urticaria showed that only 44% of patients responded well to the antihistamine treatment, of which 15% continued to show partial symptoms [25]. Owing to this, experts in the field have recommended up-dosing the drug up to fourfold in patients who do not respond satisfactorily to the standard dose treatment [1,3]. A recent meta-analysis by Xiao et al. also indicated the possibility of better outcomes with an increased antihistamine drug dose [26]. Guillén-Aguinaga et al., in their systematic review and meta-analysis, reported significant control of pruritus after up-dosing antihistamines in CSU patients [17]. However, due to a limited number of high-quality studies, it is difficult to draw conclusions from these meta-analyses. While there is insufficient literature, there are few studies that have reported improved disease control in CSU patients by increasing the daily dose of desloratadine to two to four times the prescribed 5 mg/day dose [18,27,28].

To our knowledge, this study provides the first piece of evidence in the Indian setting that in patients with moderate to severe CSU, increasing the daily dose of desloratadine to two times the standard dose of 5 mg/day improves disease outcomes, reflected by the disease severity score as well as the QoL score, without having any detrimental effect on the safety of the patients. All participants who were enrolled in the study had a history of moderate to severe CSU for at least six weeks and did not respond to the conventional daily dose of desloratadine that they had been taking for at least two weeks. We estimated the clinical severity of CSU using UAS7, which is a validated and widely used patient-reported tool recommended by the international EAACI/GA2LEN/EuroGuiDerm/APAAACI guideline for urticaria [1]. It has also been used across several clinical trials as the primary outcome measure for CSU [29-31]. It is reported in the field that the minimally important difference (MID), the minimum drop in UAS7, which is clinically meaningful, generally ranges from 9.5 to 10.5 [32]. In our study, the mean UAS7 score at baseline was 31.9 (range: 20.5-42), indicating moderate to severe CSU. After taking a high dose of desloratadine (10 mg/day) for two weeks, the mean scores dropped by 6.3 points (20% reduction) and further dropped by 13.7 points (42% reduction) after four weeks. The drop in the scores in our study was higher than the MID, indicating a statistically and clinically significant reduction in the patient-reported disease severity. The same trend was seen when participants were categorized based on their disease severity. Out of the total 225 participants who had severe CSU at baseline despite taking 5 mg/day desloratadine, 193 (85.8%) showed a reduction in severity after taking 10 mg/day desloratadine for four weeks. Only two weeks of high-dose treatment reduced the severity in 91 (40%) participants. Participants (n = 31) who had moderate CSU at baseline also showed similar improvements. In this category, disease severity showed a reduction in 20 (64.5%) participants by four weeks and in nine (29%) participants with just two weeks of treatment. Overall, 213 (83%) participants showed an improvement in their disease severity after four weeks of treatment with 10 mg/day desloratadine. These findings are in line with a study on Libyan patients that reported improvement in disease severity in 58% of CSU patients using 10 mg/day desloratadine as compared to 33% with 5 mg/day desloratadine [27]. In another study, higher-dose desloratadine had a beneficial effect on patients with cold-induced urticaria [33].

As the effects of CSU are not limited only to the physical health status of patients but also affect their emotional and social well-being, we included a tool to measure the effect of a higher-dose treatment on the QoL of patients. The CU-Q2oL was used to assess the impact of CSU on patients’ daily QoL, where a higher score indicated a more impaired QoL. It is a validated and well-accepted questionnaire to assess patients’ QoL [20]. It has been used as an assessment tool in many clinical trials [6,34,35]. A difference in the CU-Q2oL (Thai version) score of 15 has been reported as the minimal clinical important difference (MCID) that is considered a meaningful improvement by the patients [36]. In our study, the mean baseline CU-Q2oL score was 59.8, which indicated an impaired QoL. After treatment with 10 mg desloratadine daily for four weeks, the score reduced by 24.6 (38.5%), which was higher than the MCID, and the CU-Q2oL score by the end of the study was 35.4, which was statistically significantly lower than the baseline values. Thus, the clinical benefits to the participants in our study were mirrored by a substantial improvement in their QoL parameters.

A total of 15 AEs were reported (12 mild and three moderate) in 14 patients, with an incidence of 5.9%. These AEs did not differ from the usual side effects of the standard prescribed dose of the drug [22]. All the AEs were resolved during the study period. In our study, we did not find an increased incidence of somnolence. This finding is similar to that reported by Siebenhaar et al. for cold-induced urticaria patients treated with 20 mg desloratadine [33]. Henz et al.* *have reported no significant increase in sedation or drowsiness even when desloratadine is used at a dose as high as 45 mg/day [37]. Thus, overall, desloratadine was well-tolerated by the participants, which was evident from the fact that there were no withdrawals or any SAEs reported in our study.

However, there are certain limitations in this study owing to its design. As this was a single-arm, non-comparative study, the outcomes of up-dosing could not be compared with placebo or standard prescribed dose to evaluate if there were any additional benefits in disease outcome parameters. Additionally, while the national and international guidelines suggest a fourfold increase to the conventional dose, we increased the drug dose to only two times the standard dose and demonstrated that even a twofold increase in desloratadine dose improves the clinical parameters and QoL of CSU patients, without compromising their safety or cognition. Whether a higher dose of desloratadine achieves better results or the drug effect plateaus beyond the dose we used in the study needs to be evaluated through larger comparative trials.

## Conclusions

Up-dosing of desloratadine in the treatment of patients with moderate to severe CSU not responding to the licensed dose of desloratadine showed an improvement in disease severity (UAS7 and Cu-Q2oL) after four weeks of treatment. Up-dosing of desloratadine did not cause an increased incidence of somnolence and was well-tolerated. To conclude, desloratadine 10 mg could be an effective and well-tolerated treatment option for patients with moderate to severe CSU who do not respond to conventional doses of desloratadine. Further larger comparative trials can be conducted to assess the efficacy and safety of higher doses in cases where even a 10 mg dose does not provide sufficient beneficial clinical outcomes.
